# An examination of factors potentially influencing birth distributions in golden snub-nosed monkeys (*Rhinopithecus roxellana*)

**DOI:** 10.7717/peerj.2892

**Published:** 2017-01-24

**Authors:** Zuofu Xiang, Wanji Yang, Xiaoguang Qi, Hui Yao, Cyril C. Grueter, Paul A. Garber, Baoguo Li, Ming Li

**Affiliations:** 1 College of Life Science and Technology, Central South University of Forestry & Technology, Changsha, Hunan, China; 2 Key Lab of Animal Ecology and Conservation Biology, Institute of Zoology, Chinese Academy of Sciences, Beijing, China; 3 Key Lab of Conservation Biology for Shennongjia Golden Monkey, Shennongjia Forest District, Hubei, China; 4 College of Life Sciences, Northwest University, Xi’an, Shaanxi, China; 5 School of Anatomy, Physiology and Human Biology, The University of Western Australia, Crawley, WA, Australia; 6 Department of Anthropology and Program in Ecology and Evolutionary Biology, University of Illinois, Urbana, IL, United States

**Keywords:** Birth seasonality, Birth seasonality food availibility, Day length, Temperature, Golden snub-nosed monkey

## Abstract

Many species of primates are considered seasonal breeders, but the set of factors, such as food availability, day length and temperature, that influence the timing of reproductive events for both wild and captive individuals remains unclear. Here, we examine the role of factors in shaping breeding patterns in *Rhinopithecus roxellana*, a temperate colobine primate. We used circular statistics to describe and compare the patterns of reproductive seasonality among individuals in 13 captive groups and two free ranging but provisioned groups at various locations throughout China. Almost 90% of births occurred in March, April and May in adult females residing in both free ranging (*n* = 131) and captive groups (*n* = 407). Births occurred principally in 2–4 months prior to the peak of food availability, while conceptions occurred in 1–2 months after the peak of food availability in free ranging but provisioned groups. Day length (latitude) had a significant effect on the timing of reproduction. However, females that experienced a wide variation of temperature between the lowest and highest monthly average temperature had a later conception date. These results support that day length and temperature might be factor influencing the timing of reproductive activity.

## Introduction

Seasonal breeding is defined as the occurrence of births within clearly delimited periods (Van Schaik, Van Noordwijk & Nunn, 1999). It is the norm in a variety of primates living at both tropical and temperate latitudes (Di Bitetti & Janson, 2000). While documentation of patterns of reproductive seasonality and its regulation in primates have increased steadily over the past few decades, how the set of factors, such as food availability, day length and temperature, influences the timing of reproductive events in both captive and wild groups are still crucial for those endangered species.

Seasonal food availability has been cited as one of the principle factors determining seasonal breeding in primates (Di Bitetti & Janson, 2000; Brockman & Van Schaik, 2005). Given that late gestation and early lactation represent the most energetically demanding periods for reproductively active females, some primate species living in seasonally predictable environments are reported to coincide the birth season and periods of intensive nursing with increased availability or seasonal peaks in high energy-high protein foods (McCabe, Fernánd & Ehardt, 2013). This pattern of reproductive timing has been documented in some strepsirrhines (Richard et al., 2002), new world monkeys (Lewis & Kappeler, 2005b; Lewis & Kappeler, 2005a), and old world monkeys (Van Schaik, Van Noordwijk & Nunn, 1999). For example, Hanuman langurs (*Semnopithecus entellus*) give birth from January until June, and conceptions are confined to the months of July to November, coinciding with the period of highest food availability (Koenig et al., 1997). However, given that relative to body mass primates are generally characterized by an elongated period of gestation (5–9 months) and an extended period of lactation (three months to five years), female’s have evolved a range of alternative behavioral, social, and nutritional strategies designed to enhance reproductive success (Brockman & Van Schaik, 2005). Primate species such as long-tailed macaques (*Macaca fascicularis*) (Van Schaik & Van Noordwijk, 1985) and great apes (Knott, 2005), might time conception to coincide with periods of high food availability in order to store reserves that can be drawn on later during pregnancy. Other models have been proposed to explain variation in the timing of reproductive events across primate species. For example, females acting as income breeders use energy obtained throughout the reproductive period to support their developing offspring (Brockman & Van Schaik, 2005). In contrast, females acting as capital breeders are expected to time conception to periods of high food availability and use these accumulated resources during periods when the energetic costs of reproduction are high and food is less available (McCabe, Fernánd & Ehardt, 2013). For example, even in the captive condition, female Japanese Macaques (*Macaca fuscata*) still accumulate energy reserves and gain weight in fall to prepare for mating activity, and to survive the severe conditions of winter, which is the period of gestation if pregnancy occurs (Garcia, Huffman & Shimizu, 2010; Garcia et al., 2011).

Other factors, such as the natural light cycle (photoperiod), which usually is determined by the latitude and controls melatonin release and modulates circadian and circannual pacemakers (Wehr, 2001; Dardene, 2012), also can operate as a primarily proximate factor influencing the timing of reproduction (Ziegler et al., 2000; Bradshaw & Holzapfel, 2007). Some research on the effects of photoperiod in primates has focused on rhesus monkeys (*Macaca mulatta)* (Wilson & Gordon, 1989), squirrel monkeys (*Saimiri sciureus*) (Schiml et al., 1999) and tamarins (Mcgrew & Webster, 1995), tufted capuchins (Perret, 1997), and multiple species of lemurs (Carosi, Linn & Visalberghi, 2005). In these species, decreasing day length stimulates the onset of reproduction (Wilson & Gordon, 1989; Schiml et al., 1999). However, results from both laboratory studies and studies of transplanted Japanese macaques (*M. fuscata*) do not support the photoperiod hypothesis because the timing of breeding was not substantially influenced by controlled changes in exposure to different L:D cycles or when individuals were transplanted to different latitudinal zones (Nozaki, Mori & Oshima, 1990).

Evidence also suggests that temperature can influence the timing of reproduction because extremes in ambient temperature affect energy expenditure, basal metabolic rate, and the costs of thermoregulation (Dunbar, 1988). For example, Zhao & Deng (1988) found direct evidence that female Tibetan macaques (*M. thibetana*) were characterized by an earlier birth season at higher elevations (colder temperature) than at lower elevations. Similarly, based on a comparison of climatic and reproductive data among 23 provisioned groups of Japanese macaques, Cozzolino et al. (1992) found that the mean conception date was negatively related to the magnitude of the decrease in the mean temperature from summer to fall. Given that many primate species live in tropical habitats (Janson & Verdolin, 2005) in which monthly mean temperature varies only by a few degrees throughout the year and seasonal patterns of rainfall are a strong predictor of food availability (Van Schaik & Pfannes, 2005), temperature as an environmental determinant of reproductive seasonality in primates has not been fully explored (Rutherford, 1980). For primates that inhabit temperate ecosystems, changes in temperature extremes across seasons of the year are likely to have a greater effect on reproduction than food availability if the temperate species are capital breeders.

An effective methodological approach to identify factors affecting the timing of reproduction is to compare patterns of breeding seasonality between wild and captive groups of the same species. For example, if seasonal fluctuations in food availability is a primarily factor that determined seasonal breeding in the natural habitat, then animals in captivity would be expected to display a more uniform or non-seasonal breeding pattern compared to conspecifics in the wild. Furthermore, the availability of captive groups living at different latitudes and under easily monitored temperature regimes, provides a natural experiment to examine the influence of day length (latitude) and temperature variation on reproductive seasonality in primates. As of yet, the limited data available, for shortage of data from both captive and wild groups, do not allow to draw conclusions regarding the primarily factor influencing breeding activities of a colobine species, the foregut-fermenting primates for which the main source of nutrition is leaves.

In this paper, we analyze and compare the reproductive patterns of adult females in 13 captive groups and two free ranging but provisioned groups of *R. roxellana. R. roxellana* are a species of endangered leaf-eating (colobine) primate endemic to China that inhabits high altitude mountainous temperate broadleaf and coniferous forests (Zhang et al., 1992). Zhang, Liang & Wang (2000) reported on the mating and birth seasonality of one captive population and suggested that births peak during the spring, a time of the year when the availability of protein-rich new leaves was assumed to be at its highest. Similarly, Qi, Li & Ji (2008) hypothesized that the pattern of birth seasonality in *R. roxellana* represented an adaptive response to the seasonality of mountain climate and food resources. Here, through using a long-term and more comprehensive data set, we test a series of hypotheses designed to examine the role of food availability, photoperiod, and temperature in influencing patterns of seasonal breeding and birth seasonality in *R. roxellana*: (1) If food abundance is a primary factor in determining the likelihood of conception, captive animals given nutritionally adequate diets should fail to exhibit seasonal variation in breeding; we thus predict a significant difference in breeding patterns between free ranging and captive groups at similar latitude; (2) If mating and fertility are tied to photoperiod, then we expect that females will conceive and give birth later in the year in groups living at more northern latitude under the same food conditions; and (3) If the magnitude of temperature variations between the coldest and the hottest month has effect the reproductive fertility in females, then we expect a later conception or birth data when there is a larger temperature variation.

## Methods

### Ethics statement

Prior to conducting this study, approval was gained from the Shennongjia National Nature Reserve (snnr-081201), the Zhouzhi National Nature Reserve (znnr-050621), and the Institutional Animal Care and Use Committee of Central South University of Forestry & Technology (csuft-090120). Data collected were purely observational in nature and were collected with minimal disturbance to the animals (although the animals were provisioned by reserve staff).

### Species

*R. roxellana* is distributed from 28°58′–33°50′N to 103°05′–110°03′E and at elevations of between 1,000 and 4,100 m above sea level in the Qionglai, Minshan, Qinling, and Daba Mountains of Central China (Zhang, Liang & Wang, 2000; Kirkpatrick & Grueter, 2010). The species is highly folivorous and its annual diet is composed principally of leaves and fruits. During the winter, when fruits and leaves are unavailable, *R. roxellana* consume a diet consisting of lichen, pine seeds, oak seeds, and bark (Kirkpatrick & Grueter, 2010; Li, 2006; Guo, Li & Watanabe, 2007). Females usually give birth once every two years provided the previous offspring survived to the age of weaning. If a female’s offspring died before 5–6 months of age, a female will give birth the following year (Qi, Li & Ji, 2008). Mating behavior was seasonal with mating peaks in September, October and November (Li & Zhao, 2007), although copulations have often been observed during other months of the year (Xiang et al., 2014).

### Birth events in the natural environment

We observed one free ranging but provisioned group of *R. roxellana* at Dalongtan (coordinates: 31°29′N, 110°18′E; elevation: 2,200 m *asl*) in Shennongjia Nature Reserve, central China. This area is a highly seasonal temperate forest. The monkeys were fed two to three times a day at different sites with lichen, pine seeds, apples, carrots, oranges, and peaches. We conducted daily observations at distances between 3 and 50 m from 0800 to 1600 in winter and spring or 0700 to 1900 in summer and autumn so long as weather permitted (Xiang et al., 2014). Seventy birth events have been accurately recorded between 2006 and 2014.

We observed the other free ranging group at Yuhuangmiao in Zhouzhi National Nature Reserve (33°46′N, 108°15′E; 1,500-2,890 m *asl*), which is located on the northern slope of the Qinling Mountains. Seasonality in temperature and food availability in this region is pronounced; the study troop is provisioned with small amounts of food 15 days per month for 6–8 months each year (Qi, Li & Ji, 2008). Sixty-one birth events have been identified between 2003 and 2006 (Guo, Li & Watanabe, 2007). Data on birth seasonality were collected through daily observations over the course of 4–6 months during the birth season in a partially provisioned group (Qi, Li & Ji, 2008).

### Birth events in the captive environment

Birth data for captive groups were provided by the Chinese Association of Zoological Gardens. The species have been maintained in more than 30 zoos and wildlife parks from Harerbin (45°45′N) in the north, to Guangzhou (23°00′) in the south of China. Almost all individuals were captured from the Qinling mountains. Animals of both sexes are kept in an outdoor enclosure during the day throughout the year but given access to an enclosed room at night. Although the night room for groups housed in northern China is usually equipped with a heating unit, we presume heating has no influence on the resumption of breeding because the heaters were not operational before mid-November. In the wildlife park, the monkeys are kept in a large enclosed area. Although captive groups have been maintained in China since 1956, and >500 births have been recorded for the total captive population, in the current study we only used birth records from 1972 because few of the birth events prior to 1972 included the exact date the infant was born. We also discarded data from sites with less than 12 birth records. A total of 407 records from 13 colonies were used in our analyses (Table 1). Data from different captive colonies within the same city, such as the Beijing Zoo and Beijing Breeding Center of Endangered Animals, and the Shanghai Zoo and Shanghai Wildlife Park, were pooled because a Watson’s *U*^2^ test showed the data were not significantly different (Batschelet, 1981).

**Table 1:** Golden snub-nosed monkeys groups, the number of snub-nosed monkey-years, and the number of births that were included in the birth and conception analyses.

Site	Latitude	Longitude	Years represented	No. of years	Birth recorder	Data resource
Free ranging groups						
Shennongjia	31°29′	110°18′	2006–2013	9	70	This study
Qingling	33°46′	108°15′	2003–2006	4	61	This study
Captive groups						
Guangzhou	23°00′	113°18′	1998–2011	14	12	CAZG^a^
Hangzhou	30°12′	120°08′	1997–2012	16	19	CAZG
Chengdu	30°42′	104°06′	1998–2012	15	18	CAZG
Tonglin	30°59′	118°02′	2000–2012	13	65	CAZG
Shanghai^b^	31°11′	121°32′	1979–2012	34	77	CAZG
Louguantai	34°03′	108°19′	1997–2010	14	19	CAZG
Xi’an	34°15′	108°59′	1981–2000	20	18	CAZG
Lanzhou	36°02′	103°49′	1984–2011	28	21	CAZG
Jinan	36°42′	116°59′	1994–2010	17	17	CAZG
Xixiakou	37°24′	122°37′	2004–2012	9	18	CAZG
Gansu ERC	37°52′	102°52′	1996–2010	15	13	CAZG
Beijing^c^	39°56′	116°20′	1972–2012	41	93	CAZG
Haerbing	45°45′	126°36′	1995–2000	6	17	CAZG
Total					538	

**Notes.**

a CAZG, Chinese Association of Zoological Gardens.

b Included records from the Shanghai Zoo and Shanghai wildlife park.

c Included records from the Beijing Zoo and Beijing Breeding Center of Endangered Animals.

### Conception events

As there are no accurate records of the precise gestation length for wild individuals, conception dates were calculated by deducting 202 days from birth dates; 202 days (*sd* = 1.1 days, *n* = 5) is the average gestation of captive individuals (Yan et al., 2003; Qi et al., 1995).

### Food availability in the natural environment

For the Shennongjia group, the availability of young/mature leaves, flowers, and fruits/seeds follows a distinctly seasonal pattern; young leaves appeared from April to August, mature leaves from May to September, flowers from May to June, and fruits/seeds from July to October; lichens and buds were almost year-round available with active buds from April to August and dormant buds from September to next March (Qi, Li & Ji, 2008). A similar pattern of food availability was found for the Qinling group (Guo, Li & Watanabe, 2007). Given that captive groups are generally fed the same diet throughout the year, with only slight variation based on seasonal availability of fruits in local stores, we assumed that *R. roxellana* housed in different zoos and wildlife parks were fed a nutritionally adequate die and much less seasonal compared with the ones in the natural environment. This is supported by the fact that there were no difference in female’s reproductive parameters, such as inter-birth interval (*t*_41,289_ = 0.621, *p* < 0.05) and age at first reproduction (*t*_8,73_ =  − 1.53, *p* > 0.05) between free ranging and captive groups (See Supplemental Information).

### Photoperiod

As the photoperiod was usually determined by the latitude, therefore the latitude was used as substitution for photoperiod in the data analysis.

### Temperature

There are many meteorological stations maintained by the National Meteorological Bureau of China. We used the temperature data provided by the nearest National Meteorological Bureau of China station to the zoos or wildlife parks for the study (Table 2). The following temperature data were included in this analysis: mean annual temperature, mean highest temperature, highest monthly temperature, the temperature variation between the lowest and highest monthly average temperature, and the largest variation in daily mean temperature during one and two months (absolute value of temperature decrease in one and two months) immediately following the month with the highest mean monthly temperature. We decided to examine variation in temperature in one and two months following the highest monthly temperatures because conceptions in *R. roxellana* occur in early fall, approximately two months after the highest temperatures of the summer. We decided to examine the temperature variation between the lowest and highest monthly average temperature because we want to test if energetic stress has affected the reproduction, as extreme temperature will be effect the expenditure of energy of an animal.

**Table 2:** Mean annual and monthly temperatures (°C) at two field sites and 13 Zoos and wildlife parks.

Site	Annual	Jan	Feb	Mar	Apr	May	Jun	Jul	Aug	Sep	Oct	Nov	Dec
Guanzhou	22.1	13.6	14.6	17.9	22.1	25.5	27.6	28.6	28.4	27.2	24.2	19.6	15.3
Hangzhou	16.5	4.3	5.7	9.6	15.8	20.7	24.4	28.4	27.9	23.4	18.3	12.4	6.8
Chengdu	16.1	5.6	7.5	11.5	16.7	21	23.7	25.2	25	21.2	17	12.1	7.2
Tonglin	16.7	4	5.8	10	16.6	21.8	25.3	28.7	28.4	23.7	18.3	12.1	6.4
Shanghai	16.1	4.2	5.3	8.8	14.6	19.6	23.8	27.9	27.7	23.7	18.7	12.7	6.6
Shennongjia	7.1	−3.5	−2.1	2.3	8.8	12.3	15.4	17.1	16.3	12.5	8.6	1.5	−1.8
Qingling	10.7	0.2	1.9	6.1	12.2	15.9	19.1	21.2	21	16.1	10.8	6.2	1.7
Louguantai	11.6	0.7	2.5	6.6	12.8	16.5	19.7	21.8	21.5	16.6	11.7	6.7	2.2
Xi’an	13.9	−0.1	2.9	8.1	14.8	19.8	24.8	26.6	25.3	19.9	13.9	6.9	1.3
Lanzhou	9.8	−5.3	−1	5.4	12.2	17	20.4	22.4	21.2	16.3	9.8	2.5	−3.9
Jinan	14.7	−0.4	2.2	8.2	16.2	21.8	26.4	27.5	26.3	22	16.1	8.3	1.8
Xixiakou	12.5	−2.9	−0.5	5.5	13.1	18.9	23.7	26.2	25.2	20.5	14.2	6.3	−0.3
Gansu ERC	7.9	−7.8	−4.2	2.5	10.4	15.7	19.3	21.5	20.4	14.9	7.8	0.2	−5.8
Beijing	12.3	−3.7	−0.7	5.8	14.2	19.9	24.4	26.2	24.9	20	13.1	4.6	−1.5
Haerbin	5.7	−15.1	−10.7	−2	7.9	15.3	20.6	23.1	21.6	15.4	7	−3.4	−11.8

### Statistical analysis

We used circular statistics (Li & Zhao, 2007) to describe and compare the degree of differences in reproductive seasonality. Daily or seasonal frequency patterns typically have a circular distribution and these cyclic events may be represented by a rotation of 360 degrees (Li & Zhao, 2007). When describing the seasonality of births, the total length of the circular axis is the year, creating an axis divided into 365 equal sections, where each sector is 360°/365. Each day, coded using the Julian calendar, is then converted to an angle that divides the circle into equal sections (Li & Zhao, 2007). Each reproductive event is converted to a vector with an angle, *α*, and a length, *γ*. Trigonometric functions were used to analyze all vectors (Guo, Li & Watanabe, 2007), resulting in a mean vector angle (*μ*) and a mean vector length (*γ*). The mean angle can be converted back to a mean date. The mean vector length of *γ* gives an indication of how widely spaced the observations are on the axis, with values ranging from 0 to 1.0, where 0 means that all observations occurred during the same interval (Li & Zhao, 2007). Raleigh’s uniformity test was used to evaluate whether the birth and (estimated) conception date were distributed uniformly on the circular axis (Batschelet, 1981).

To analyze if food availability has influenced reproductive events when controlling for the effect of latitude, we compared the reproductive patterns of free ranging but provisioned groups to the captive colonies. In this analysis, the Watson’s *U*^2^ test was used to determine whether the degree of variability in the timing of conceptions (estimated conception date) among females in the free ranging but provisioned group differed from females in the captive groups (Batschelet, 1981) in terms to test the probability that two samples of circular data come from the same population.

Spearman’s rho correlation was used to determine the relationship between mean conception date and latitude. To control for the influence of food availability on reproductive events, we performed analyses with and without the free ranging group.

Spearman’s rho correlation also was used to analyze the relationship between the mean date of conception and variation in daily temperature. To control for the influence of food availability on reproductive events, we also performed analyses with and without the free ranging group.

The circular statistical program *Oriana verion 4* (Kovach, 2011) was used to analyze evidence of differences in reproductive seasonality and the mean and median date of conceptions and births. Spearman’s rho correlations were analyzed using SPSS 13.0. Statistical tests were two-tailed and probability was set at 0.05.

## Results

### Reproductive seasonality of two free ranging groups

**Figure 1:**
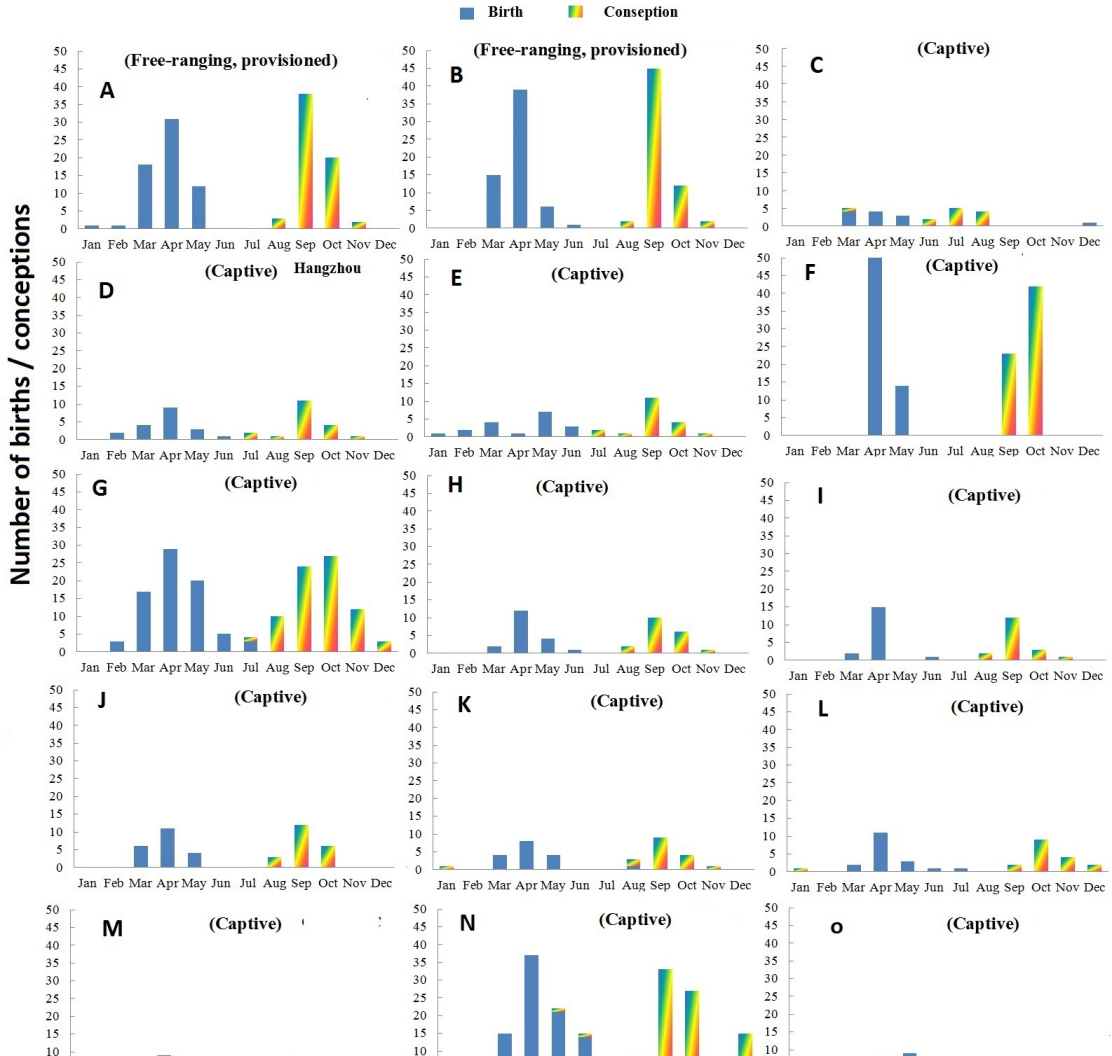
Distribution of births and conceptions in two free ranging groups (A–B) and 13 zoos/wildlife parks (C–O) in China. (A) Shennongjia; (B) Qingling; (C) Guangzhou; (D) Hangzhou; (E) Chengdu; (F) Tonglin; (G) SHanghai; (H) Louguantai; (I) Xi’an; (J) Lanzhou; (K) Jinan; (L) Xiaxiakou; (M) Gansu ERC; (N) Beijing; (O) Haerbing.

Births and extrapolated conception events of two groups of *R. roxellana* inhabiting in the natural environment are presented in Fig. 1. Of all birth events in the two wild groups, 97.7% took place between March and May.

The records of Shennongjia group include 70 births involving 26 adult females that occurred over a nine-year period. The frequency of birth and conception in Shennongjia differed significantly from a random distribution (Rayleigh test, *p* < 0.001, Table 3). Nearly half of birth events occurred in April; and together with those that occurred in March (30.0%) and May (21.4%), 98.6% births occurred in a three month period (Fig. 1) except one premature birth on January 29, 2011 and one normal birth on February 26, 2010. Both the mean birth date and median birth date were April 16. Conception occurred in August (4.3%), September (60.0%), October (31.4%) and November (4.3%) (Fig. 1). Both the mean and median conception date was September 26.

**Table 3:** Birth, (estimated) conception distribution and circular statistics for the births at 15 sites (13 captive and two free-ranging) in China that had more than 12 births recorded.

	Birth					Conception										
Sites	Mean vector (*μ*)	Mean group	Length of mean vector (*r*)	Median	Median group	Mean vector (*μ*)	Mean group	Length of mean vector (*r*)	Median	Median group	Concen tration	Circular variance	Circular standard deviation	Standard error of mean	Rayleigh test (*Z*)	Rayleigh test (*p*)
Free-ranging groups																
Shennongjia	104.203	106	0.953	104.055	106	264.97	269	0.953	264.822	269	10.856	0.047	17.822	2.245	57.19	<0.001
Qingling	101.37	103	0.967	101.096	103	262.14	266	0.967	261.863	266	15.595	0.033	14.754	1.889	57.086	<0.001
Captive groups																
Guangzhou	97.11	99	0.814	105.041	107	257.88	262	0.814	265.808	270	2.324	0.186	36.804	12.03	7.943	<0.001
Hangzhou	97.485	99	0.901	96.164	98	258.25	262	0.901	256.932	261	5.357	0.099	26.118	5.982	15.435	<0.001
Chengdu	101.476	103	0.738	118.356	121	262.24	266	0.738	279.123	284	2.263	0.262	44.709	10.45	9.791	<0.001
Tongling	112.517	115	0.991	113.918	116	273.28	278	0.991	274.685	279	56.424	0.009	7.662	0.95	63.848	<0.001
Shanghai	111.886	114	0.864	111.945	114	272.65	277	0.864	272.712	277	3.981	0.136	30.993	3.521	57.466	<0.001
Louguangtai	106.349	108	0.943	103.068	105	267.12	271	0.943	263.836	268	8.974	0.057	19.712	4.52	16.879	<0.001
Xi’an	97.935	100	0.956	92.219	94	258.7	263	0.956	252.986	257	11.52	0.044	17.275	4.07	16.436	<0.001
Lanzhou	97.574	99	0.955	99.123	101	258.34	262	0.955	259.89	264	11.386	0.045	17.381	3.792	19.154	<0.001
Jinan	102.446	104	0.857	92.219	94	263.21	267	0.857	252.986	257	3.819	0.143	31.773	7.679	12.499	<0.001
Xixiakou	141.222	144	0.896	133.151	135	302.05	307	0.896	293.918	298	5.117	0.104	26.803	6.306	14.462	<0.001
Gansu ERC	116.927	119	0.935	105.041	107	277.69	282	0.935	265.808	270	6.196	0.065	21.069	6.604	11.356	<0.001
Beijing	113.126	115	0.778	108.986	111	273.89	278	0.778	269.753	274	2.615	0.222	40.632	4.166	56.243	<0.001
Haerbing	116.254	118	0.934	113.918	116	277.02	281	0.934	274.685	279	7.816	0.066	21.225	5.144	14.82	<0.001

The data of Qinling group include 61 births involving 42 adult females that occurred over a four year period. The frequency of births and conceptions differed significantly from a random distribution (Rayleigh test, *p* < 0.001, Table 3). Most births occurred in March (24.6%), April (63.9%) and May (9.8%), with one birth occurring in June 2005 (Fig. 1). Both the mean birth date and median birth date were April 13. Conceptions occurred in August (3.2%), September (73.8%), October (19.8%) and November (3.2%) (Fig. 1). Both the mean and median conception dates were September 23.

### Reproductive seasonality of captive groups

Births and extrapolated conception events of 151 females living in 13 captive groups are presented in Fig. 1. Overall, the number of total births recorded was 407. The frequency of births and conceptions differed significantly from a random distribution (Rayleigh test: *p* < 0.001, Table 3). There was virtually no overlap in birth and conception dates.

In captivity, births occurred between February and June with 88.1% of births in March (14.8%), April (50.4%) and May (22.9%). The only exceptions involved one infant born on December 9, 1998 in the Beijing Zoo and one infant born on December 15, 2009 in Guangdong. Both infants died within a day of their birth. The mean birth date for the captive population was April 20 and the median birth date was April 18.

In the captive groups, 83.2% of conceptions occurred in August (8.6%), September (38.3%) and October (36.3%). The mean conception date was October 2 and the median conception date was October 1.

### Factors associated with birth seasonality

#### Food availability

For the free ranging but provisioned groups, births occurred principally in the spring 2–4 months prior to the peak of food availability, while conceptions occurred in the late Fall 1–2 months after the peak of food availability (Li, 2006; Guo, Li & Watanabe, 2007). All captive groups were also found to be strictly seasonal breeders despite the fact that the quality and quantity of food provided across the year was relatively constant. There was no significant difference in reproductive patterns between two free ranging but provisioned groups and the captive groups (Watson’s *U*^2^ test, *U* = 0.135, *p* > 0.05).

#### Photoperiod

**Figure 2:**
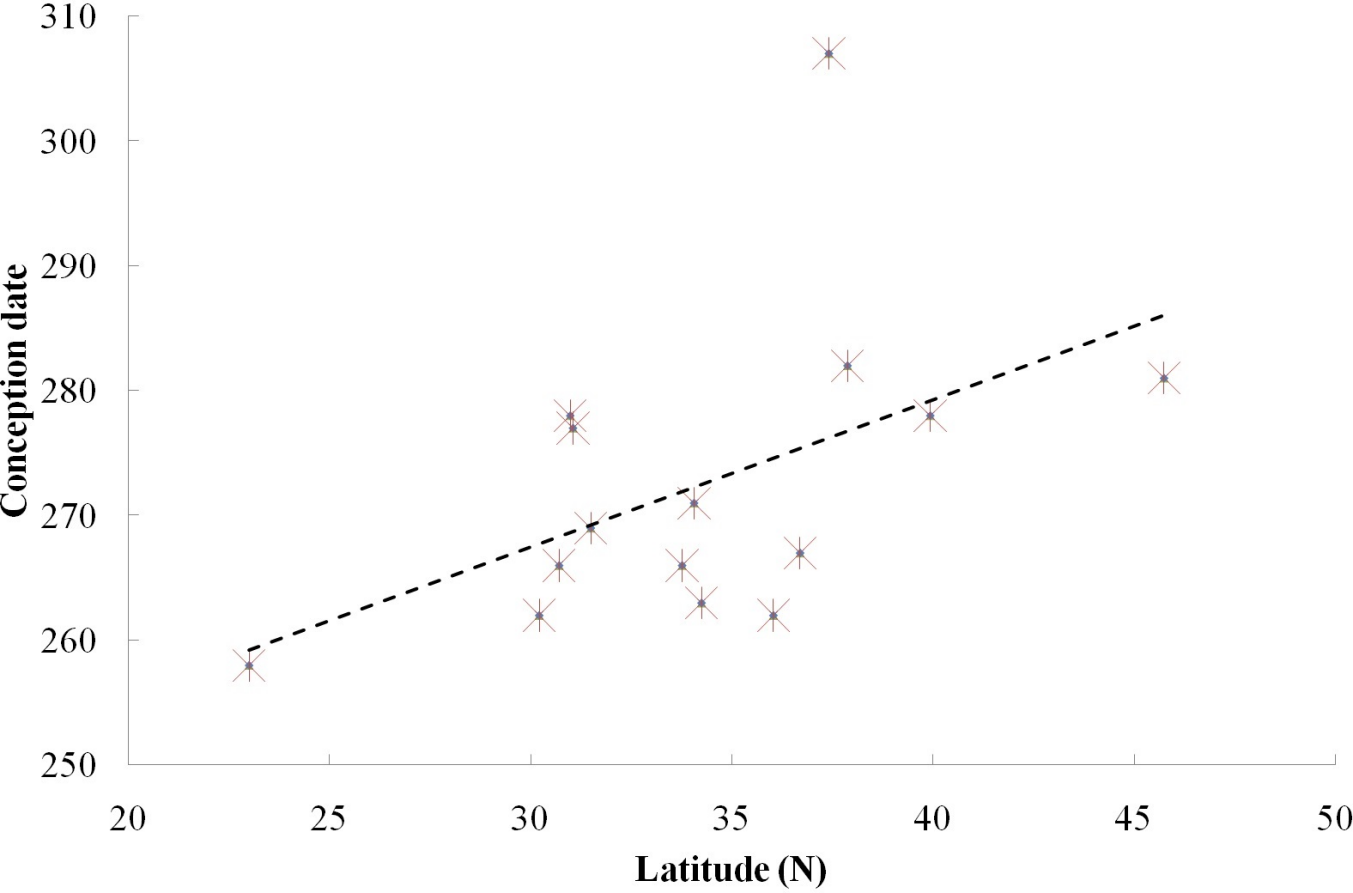
Relationship between reproductive events (conception date) and latitude at two field sites and 13 zoos/wildlife parks in China.

We found a significant relationship between the timing of conceptions and latitude among the 15 locations of the 13 captive groups and two free ranging groups (Fig. 2, Spearman correlation test, *r*_*s*_ = 0.669, *p* < 0.05). Furthermore, when we excluded the free ranging groups, there still was a significant relationship between the timing of reproductive events and latitude among the captive population (*r*_*s*_ = 0.603, *p* < 0.05), which means that females conceive later in the year in groups living at higher latitude under the same food conditions.

#### Temperature

**Figure 3:**
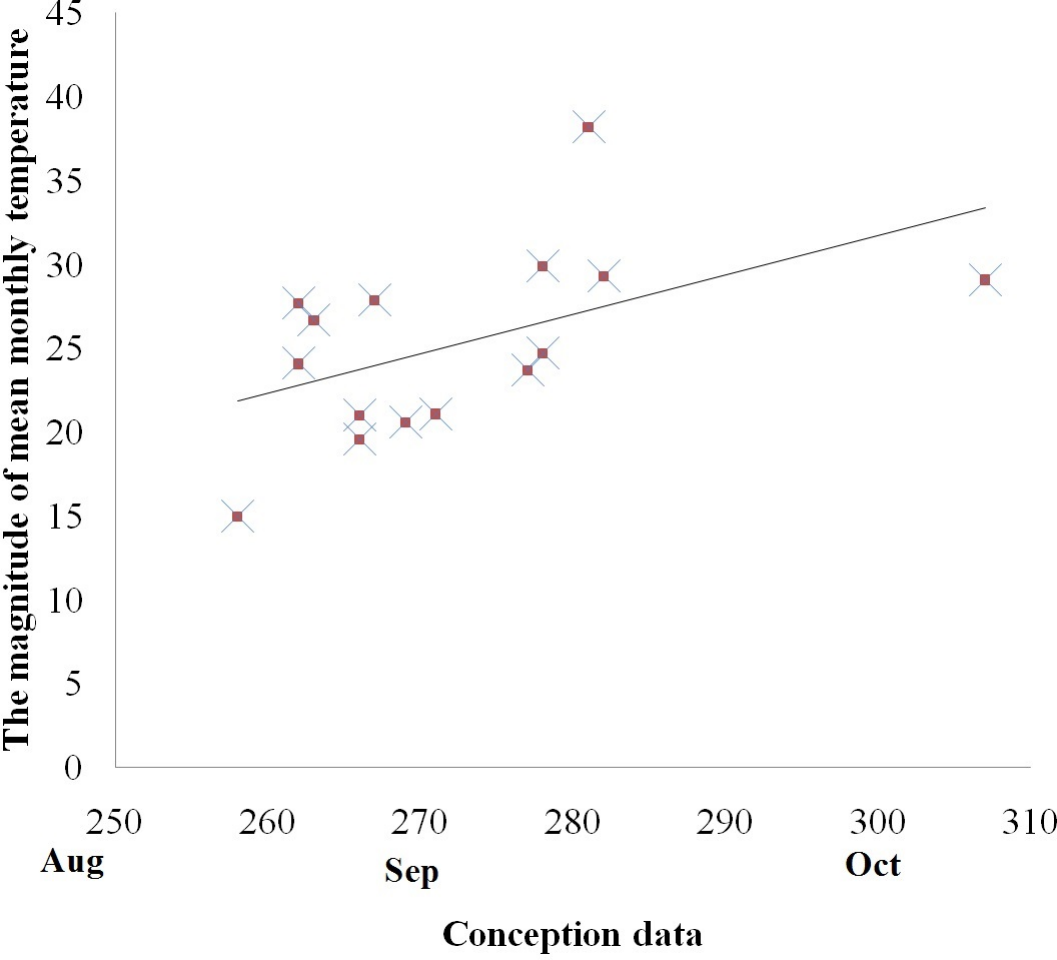
Relationship between the magnitude of mean monthly temperature between the coldest and the hottest month and reproductive events (conception) among golden snub-nosed females inhabiting two field sites and 13 zoos/wildlife parks in China. *X* axis, conception date in the Julian calendar; *Y* axis, the magnitude of mean monthly temperature.

There was, however, a positive correlation between the mean conception date and the magnitude of temperature variations between the coldest and the hottest month in both the captive and free ranging groups (Spearman correlation, with free ranging groups: *r*_*s*_ = 0.578, *p* < 0.05, Fig. 3; without free ranging groups: *r*_*s*_ = 0.620, *p* < 0.05). No other associations were identified between the mean conception date and other temperature variables such as mean annual temperature, mean highest temperature, highest monthly temperature, the magnitude of temperature decrease in the month following the hottest month of the year, and the magnitude of temperature decrease in the two months following the hottest month of the year.

## Discussion

Using long-term birth data, we analyzed the characteristics of reproductive patterns in captive and free ranging but provisioned groups of *R. roxellana*. Using the definition of 66.7% of births occurring within a three month period as an indication of breeding seasonally in primates (Van Schaik, Van Noordwijk & Nunn, 1999), *R. roxellana* is best described as a highly seasonal breeder, with >90% of births occurring between March and May. Furthermore, using circular statistics, we analyzed the relationship between reproductive seasonality (in terms of mean conception date distributions) and environmental factors, such as food availability, day length (latitude) and temperature. The results indicated that latitude had effect on the timing of reproductive events. However, marked variation in monthly temperature (absolute value of temperature increase) from the coldest month (usually in January) to the peak highest temperature which occurred during the month of July, had the strongest effect on the timing of reproductive activity in both wild and captive groups of *R. roxellana* inhabiting temperate latitudes.

Although several studies report variable reproductive patterns in wild primates, primate reproduction tends to be less seasonal in captive environments. For example, spider monkeys (*Ateles* spp.) are non-seasonal breeders in captivity, but exhibit seasonal reproduction in the wild (Chapman & Chapman, 1990). In contrast, captive brown capuchin monkeys (*Cebus apella*) remain seasonal breeders even when they are provisioned (Bicca-Marques & Gomes, 2005). When one group of rhesus macaques was shifted from Cayo Santiago Island, Puerto Rico to the German Primate Centre, they displayed a non-seasonal reproductive pattern, but returned to seasonal breeding after experiencing constant temperatures and day-length periods for four years (Kaumanns, Singh & Schwibbe, 2013). In the current study, we found that *R. roxellana* remains a strictly seasonal breeder in both captive and free ranging environments despite living at different latitudes, having access to differences in food availability and predictability, and exposed to different temperature regimes.

In primates, seasonal breeding is particularly common in species inhabiting higher latitudes or altitudes, where food availability exhibits pronounced seasonal variation (Di Bitetti & Janson, 2000; Brockman & Van Schaik, 2005). In their natural environment, *R. roxellana* mainly give birth during March through May, which is 2–4 months prior to the short period of highest food availability reported to occur at two field sites, Shennongjia (Li, 2006), and Qinling (Guo, Li & Watanabe, 2007), where *R. roxellana* have been studied. This result is also in keeping with the birth seasonality reported in *R. bieti* (Cui et al., 2006; Xiang & Sayers, 2009), *Cebus capucinus* (Carnegie, Fedigan & Melin, 2011), *Colobus badius* (Struhsaker, 1975), *Semnopithecus entellus* (Lewis & Kappeler, 2005b), *Trachypithecus pileata* (Stanford, 1991), *Procolobus verus* (Korstjens, 2001) and *Macaca assamensis* (Heesen et al., 2013). However, the period of weaning in *R. roxellana* is adjusted to a period of high food availability (Stanford, 1991). In March, spring buds and young leaves begin to flush and therefore provide high protein foods for weaned infants/young juveniles. During the weaning, mothers also build up energy reserves over the spring and summer to get into reproductive condition reproduction and ovulate at the end of the summer and early fall; this also provide an explanation for the later birth and conception peaks at higher latitude. Therefore, they fit a model of a capital breeder and use accumulated resources for gestation. Thus, to increase their fitness, the long-lived and high altitude resident *R. roxellana* adopted an optimal reproductive strategy for both infant and maternal survival by prolonging their reproductive intervals to more than two years. This strategy take the advantage of both capital and income breeder. The same reproductive are also reported in both captive and natural population of *R. bieti* (Cui et al., 2006; Xiang & Sayers, 2009).

All captive groups of *R. roxellana* remained highly seasonal breeders even though the individuals were fed nutritionally adequate diets and experienced no seasonal changes in food availability. These results imply that variation in food availability may not be the primary factor that initiates reproduction in this primate species. *R. roxellana* is highly folivorous with leaves and lichens forming the major dietary constituents throughout the year, supplemented with flowers, seeds/fruits, ubiquitous buds, and bark (Li, 2006; Guo, Li & Watanabe, 2007). Food items like buds, leaves and lichens are less seasonal than fruits and seeds, and provide the monkeys with a rich source of protein and structural carbohydrates (Liu et al., 2013; Clutton-Brock, Albon & Guiness, 1989) to support one of the most energy-demanding phases of the reproductive cycle, late pregnancy and lactation (Heesen et al., 2013). Secondly, the average female body weight is sufficiently large, at 9.4 kg (Kirkpatrick & Grueter, 2010), to buffer against fluctuations in food availability (Wehr, 2001). Di Bitetti & Janson (2000) found that the degree of seasonality of reproduction in new world monkeys was related to diet, latitude, and body size. They suggested that more folivorous species were less reproductive seasonality than frugivores and insectivores. Small-bodied species, which have shorter life spans and are therefore subject to time constraints, have to confine their reproduction to a part of the year, while large-bodied species are buffered against variation in food availability. The diet and life history may affect the timing of births in large-bodied platyrrhines under the same seasonal ecological conditions (Strier, Mendes & Santos, 2001).

Our results did support the hypothesis that the timing of reproduction was regulated by seasonal changes in photoperiod as the females conceived and gave birth later in the year in groups living at more northern latitude. However, Nozaki, Mori & Oshima’s (1990) findings on Japanese macaques in laboratory and transplanted groups did not support this hypothesis. This suggests that the photoperiod has different effect on triggering seasonal breeding in *R. roxellana* and Japanese macaques, even though several anatomical and molecular studies imply that the neural-hormonal mechanisms associated with photoperiod does affect seasonality of reproduction in monkeys and humans (Wehr, 2001).

The noteworthy result in this study is that female *R. roxellana* experiencing a higher temperature variations between the coldest and the hottest month had a later mean conception and birth date. The possible mechanism through which temperature might influence reproductive activity is the energetic stress hypothesis, which proposes that energetic stress associated with the costs of thermoregulation can suppress or delay reproduction to conserve future reproductive efforts for a time when the environment is more favorable (Wasser, 1996) or females may need longer to reach critical body condition for ovulation at larger variation of temperature. Females usually conceive when they are able to increase fat stores and/or energetic stress is the least as females allocate energy to reproduction once their body is in positive energy balance or has low daily maintenance costs in Japanese macaques, apes and human (Garcia, Huffman & Shimizu, 2010; Garcia et al., 2011; Roenneberg & Aschoff, 1990; Bronson, 1995; Ellison, Valeggia & Sherry, 2005).

In temperate ecosystem, energetic stress might include nutrient deficiencies during the time that shortage of high quality food as well as costs associated with thermoregulation. Therefore, except food availability, the other possible mechanism that initiate breeding seasonality through energetic stress might be temperature. There are four processes that mainly determine the expenditure of energy of an animal: basal metabolic rate (BMR), active metabolism, growth and reproduction (Carosi, Linn & Visalberghi, 2005). BMR also is partly determined by the costs of thermoregulation, i.e., when the ambient temperature drops substantially below normal body temperature, energy has to be expended for homeostasis. For example, Nakayama et al. (1971) reported that the energy expenditure of outdoor-living captive Japanese macaques at 5.2 °C is 2.5 times larger than that at 29.5 °C. For study groups, mean monthly temperatures in July average is 25.3 °C, while in January it decreases to −0.41 °C. Although photoperiod duration, food abundance and temperature show annual variation in the temperate forests, ambient temperature may be more directly related to the animal’s energy expenditure for the costs of thermoregulation. Considering the variation in temperature and food abundance, the higher temperature variations between the coldest and the hottest month might mean higher energetic stress, which means that *R. roxellana* females may need more time to reach a good body condition to initiate hormonal changes and regulate fertility. Therefore, if an animal endures a higher temperature variation of environmental temperature would result a later conception or birth.

Although many studies have investigated the relationship between female body condition and conception (Van Schaik, Van Noordwijk & Nunn, 1999; Lewis & Kappeler, 2005b; Van Schaik & Van Noordwijk, 1985; Emery Thompson & Wrangham, 2008), only a few studies (Lewis & Kappeler, 2005a; Koenig et al., 1997; Emery Thompson & Wrangham, 2008; Bercovitch, 1987) have relied on direct evidence, such as measures of body weight or adiposity. Recently, the mechanisms regulating primate reproductive energetic has been examined in wild Sanje mangabeys (*Cercocebus sanjei*) (McCabe & Emery Thompson, 2013) and chimpanzees(*Pan troglodytes*) (Emery Thompson, Muller & Wrangham, 2012) by measuring C-peptide as an indicator of energy balance. In the future, new methodologies should be used to measure reproductive energetics, body fat, C-peptide and other indicators of energy storage and energy balance in *R. roxellana*.

Black and white snub-nosed monkeys (*R. bieti*) in high altitudes and temperate forests also have a seasonal reproductive pattern. Conception coincides with the end of the period of highest temperatures and food availability, and births occur during periods of increasing temperatures and food availability (Xiang & Sayers, 2009; Huang et al., 2012), suggesting that at least one environmental factor (temperature and/or food availability) controls the timing of conception. By examining captive data, Xiang & Sayers (2009) speculated that temperature might be a critical factor explaining the degree of differences in birth seasonality between wild and captive groups in this species. Similar patterns of seasonal reproduction have also been observed in Hanuman langurs, which occur throughout the Indian subcontinent and Sri Lanka (Lewis & Kappeler, 2005b), and in a group of wild rhesus macaques that reside in the harshest habitats of northern China (Tian et al., 2013). Considering that temperature and resource variation also influence the reproductive pattern of Japanese Macaques (Nozaki, Mori & Oshima, 1990) and Tibetan Macaques (Zhao & Deng, 1988), we argue that the reproductive energetic stress hypothesis may apply to all temperate non-human primates.

The seasonal reproduction of *R. roxellana* is a complex adaptive response to its extreme high altitude temperate habitat (1,000–4,100 m *asl*) characterized by low food productivity during late fall and winter (November to March). Reproduction in the wild may be determined by multiple factors. Food abundance and nutrient availability play a crucial role; lower quality and quantity of food availability in the winter as well as ambient temperatures below 0 °C may put an energetic stress on both males and females. Food availability might have on the breeding activity might in a different extend when considered there is different influence of food availability on reproductive patterns between free ranging group and those captive group in similar latitude. Generally speaking, in the temperate and tropical zones, the availability of foods such as fruits, leaves, and insects vary with ambient temperature although other foods such as lichens, was available year round. Our data from captive colonies provide a unique opportunity to distinguish the effects of temperature from variation of food availability in the timing of reproduction; however, more physiological research is required to confirm the hypothesis that energetic stress by temperature and food availability initiate the breeding of temperate primate.

##  Supplemental Information

10.7717/peerj.2892/supp-1 Data S1 Dataset All the observed birthdates for captive groups in zoos and wildlife parks; Dataset used for the Figs. 1, 2 and 3.

## Additional Information and Declarations

### Field Study Permissions

The following information was supplied relating to field study approvals (i.e., approving body and any reference numbers): The Shennongjia National Nature Reserve (snnr-081201), the Zhouzhi National Nature Reserve (znnr-050621).

### Data Availability

The following information was supplied regarding data availability: The raw data was provided as Data S1.

## References

[ref-1] Batschelet E (1981). Circular statistics in biology.

[ref-2] Bercovitch FB (1987). Female weight and reproductive condition in a population of olive baboons (*Papio anubis*). American Journal of Primatology.

[ref-3] Bicca-Marques JC, Gomes DF (2005). Birth seasonality of *Cebus paella* (Platyrrhini, Cebidae) in Brazilian zoos along a latitudinal gradient. American Journal of Primatology.

[ref-4] Bradshaw WE, Holzapfel CM (2007). Evolution of animal photoperiodism. Annual Review of Ecology, Evolution, and Systematics.

[ref-5] Brockman DK, Van Schaik CP, Brockman DK, Van Schaik CP (2005). Seasonality and reproductive function. Seasonality in primates: studies of living and extinct human and nonhuman primates.

[ref-6] Bronson FH (1995). Seasonal variation in human reproduction: environmental factors. Quarterly Review of Biology.

[ref-7] Carnegie SD, Fedigan LM, Melin AD (2011). Reproductive seasonality in female capuchins (*Cebus capucinus*) in Santa Rosa (Area de Conservación Guanacaste), Costa Rica. International Journal of Primatology.

[ref-8] Carosi M, Linn GS, Visalberghi E (2005). The sexual behavior and breeding system of tufted capuchin monkeys (*Cebus apella*). Advances in the Study of Behavior.

[ref-9] Chapman CA, Chapman LJ (1990). Reproductive biology of captive and free-ranging spider monkeys. Zoo Biology.

[ref-10] Clutton-Brock TH, Albon SD, Guiness FE (1989). Fitness costs of gestation and lactation in wild mammals. Nature.

[ref-11] Cozzolino R, Cordischi C, Aureli F, Scucchi S (1992). Environmental temperature and reproductive seasonality in Japanese macaques (*Macaca fuscata*). Primates.

[ref-12] Cui LW, Ai HS, He SC, Xiao W (2006). Birth seasonality and interbirth interval of capative *Rhinopithecus bieti*. American Journal of Primatology.

[ref-13] Dardene H (2012). Melatonin-dependent timing of seasonal reproduction by the Pars tuberalis: pivotal roles for long daylengths and thyroid hormones. Journal of Neuroendocrinology.

[ref-14] Di Bitetti MS, Janson CH (2000). When will the stork arrive? Patterns of birth seasonality in neotropical primates. American Journal of Primatology.

[ref-15] Dunbar RIM (1988). Primate social systems.

[ref-16] Ellison PT, Valeggia CR, Sherry DS, Brockman DK, Van Schaik CP (2005). Human birth seasonality. Seasonality in primates: studies of living and extinct human and nonhuman primates.

[ref-17] Emery Thompson M, Muller MN, Wrangham RW (2012). The energetics of lactation and the return to fecundity in wild chimpanzees. Behavioral Ecology.

[ref-18] Emery Thompson M, Wrangham RW (2008). Diet and reproductive function in wild female chimpanzees (*Pan troglodytes schweinfurthii*) at Kibale National Park, Uganda. American Journal of Physical Anthropology.

[ref-19] Garcia C, Huffman M, Shimizu K (2010). Seasonal and reproductive variation in body condition in captive female Japanese macaques (*Macaca fuscata*). American Journal of Primatology.

[ref-20] Garcia C, Huffman MA, Shimizu K, Speakman JR (2011). Energetic consequences of seasonal breeding in female Japanese macaques (*Macaca fuscata*). American Journal of Physical Anthropology.

[ref-21] Guo ST, Li BG, Watanabe K (2007). Diet and activity budget of *Rhinopithecus roxellana* in the Qinling Mountains, China. Primates.

[ref-22] Heesen M, Rogahn S, Ostner J, Schülke O (2013). Food abundance affects energy intake and reproduction in frugivorous female Assamese macaques. Behavioral Ecology and Sociobiology.

[ref-23] Huang Z, Cui L, Scott MB, Wang S, Xiao W (2012). Seasonality of reproduction of wild black-and-white snub-nosed monkeys (*Rhinopithecus bieti*) at Mt. Lasha, Yunnan, China. Primates.

[ref-24] Janson C, Verdolin J, Brockman DK, Van Schaik CP (2005). Seasonality of primate births in relation to climate. Seasonality in primates: studies of living and extinct human and nonhuman primates.

[ref-25] Kaumanns W, Singh M, Schwibbe M (2013). Environmental changes and housing conditions result in disappearance and return of reproductive seasonality in rhesus macaques (*Macaca mulatta*). Current Science.

[ref-26] Kirkpatrick RC, Grueter CC (2010). Snub-nosed monkeys: multilevel societies across varied environments. Evolutionary Anthropology.

[ref-27] Knott CD, Brockman DK, Van Schaik CP (2005). Energetic responses to food availability in the great apes: implications for hominid evolution. Seasonality in primates: studies of living and extinct human and nonhuman primates.

[ref-28] Koenig A, Borries C, Chalise MK, Winkler P (1997). Ecology, nutrition, and timing of reproductive events in an Asian primate, the Hanuman langur (*Presbytis entellus*). Journal of Zoology.

[ref-29] Korstjens AH (2001). The mob, the secret sorority, and the phantoms: an analysis of the socio-ecological strategies of the three colobines of Taï. PhD thesis.

[ref-30] Kovach WL (2011).

[ref-31] Lewis RJ, Kappeler PM (2005a). Are Kirindy sifaka capital or income breeders? It depends. American Journal of Primatology.

[ref-32] Lewis RJ, Kappeler PM (2005b). Seasonality, body condition and the timing of reproduction in *Propithecus verreauxi verreauxiin* the Kirindy Forest. American Journal of Primatology.

[ref-33] Li YM (2006). Seasonal variation of diet and food availability in a group of Sichuan snu-nosed monkeys in Shennongjia Nature Reserve, China. American Journal of Primatology.

[ref-34] Li BG, Zhao DP (2007). Copulation behavior within one-male groups of wild *Rhinopithecus roxellana* in the Qinling Mountains of China. Primates.

[ref-35] Liu X, Stanford CB, Yang J, Yao H, Li Y (2013). Foods eaten by the sichuan snub-nosed monkey (*Rhinopithecus roxellana*) in shennongjia national nature reserve, China, in relation to nutritional chemistry. American Journal of Primatology.

[ref-36] McCabe GM, Emery Thompson M (2013). Reproductive seasonality in wild Sanje mangabeys (*Cercocebus sanjei*), Tanzania: relationship between the capital breeding strategy and infant survival. Behaviour.

[ref-37] McCabe GM, Fernánd D, Ehardt CL (2013). Ecology of reproduction in Sanje Mangabeys (*Cercocebus sanjei*): dietary strategies and energetic condition during a high fruit period. American Journal of Primatology.

[ref-38] Mcgrew WC, Webster JP (1995). Birth seasonality in cotton-top tamarin (*Saguinus oedipus*) despite constant food supply and body weight. Primates.

[ref-39] Nakayama T, Hori T, Nagasaka T, Tokura H, Tadaki E (1971). Thermal and metabolic response in the Japanese monkey at temperatures of 5–38 °C. Journal of Applied Physiology.

[ref-40] Nozaki M, Mori Y, Oshima K (1990). Effects of artificial manipulation of photoperiod on reproductive seasonality of the female Japanese monkey. The Japanese Journal of Animal Reproduction.

[ref-41] Perret M (1997). Change in photoperiodic cycle affects life span in a prosimian primate (*Microcebus murinus*). Journal of Biological Rhythms.

[ref-42] Qi XG, Li BG, Ji WH (2008). Reproductive pararmeters of wild female *Rhinopithecus roxellanan*. American Journal of Primatology.

[ref-43] Qi HJ, Liang B, Bao WY, Jia YC, Hama N, Czekala NM, Harvey NC (1995). The hormone change in the urine of female snub-nosed monkeys. Acta Theriologica Sinica.

[ref-44] Richard AF, Dewar RE, Schwartz M, Ratsirarson J (2002). Life in the slow lane? Demography and life histories of male and female sifaka (*Propithecus verreauxi verreauxi*). Journal of Zoology.

[ref-45] Roenneberg T, Aschoff J (1990). Annual rhythm of human reproduction: II. Environmental correlations. Journal of Biological Rhythms.

[ref-46] Rutherford MC (1980). Annual plant production-precipitation relations in arid and semi-arid environment. South African Journal of Science.

[ref-47] Schiml PA, Mendoza SP, Saltzman W, Lyons DM, Mason WA (1999). Annual physiological changes in individually housed squirrel monkeys (*Saimiri sciureus*). American Journal of Primatology.

[ref-48] Stanford CB (1991). The capped langur in Bangladesh: behavioral ecology and reproductive tactics.

[ref-49] Strier KB, Mendes SL, Santos RR (2001). Timing of births in sympatric brown howler monkeys (Alouatta fusca clamitans) and northern muriquis (Brachyteles arachnoides hypoxanthus). American Journal of Primatology.

[ref-50] Struhsaker TT (1975). The red colobus monkey.

[ref-51] Tian J, Wang Z, Lu J, Chen A (2013). Reproductive parameters of female *Macaca mulatta tcheliensis* in the temperate forest of Mount Taihangshan, Jiyuan, China. American Journal of Primatology.

[ref-52] Van Schaik CP, Pfannes KR, Brockman DK, Van Schaik CP (2005). Tropical climates and phenology: a primate perspective. Seasonality in primates: studies of living and extinct human and nonhuman primates.

[ref-53] Van Schaik CP, Van Noordwijk MA (1985). Interannual variability in fruit abundance and the reproductive seasonality in Sumatran long-tailed macaques (*Macaca fascicularis*). Journal of Zoology.

[ref-54] Van Schaik CP, Van Noordwijk MA, Nunn CL, Lee P (1999). Sex and social evolution in primates. Comparative primate socioecology.

[ref-55] Wasser SK (1996). Reproductive control in wild baboons measured by fecal steroids. Biology of Reproduction.

[ref-56] Wehr TA (2001). Photoperiodism in human and other primates: evidence and implications. Journal of Biological Rhythms.

[ref-57] Wilson ME, Gordon TP (1989). Nocturnal changes in serum melatonin during female puberty in rhesus monkeys: a longitudinal study. Journal of Endocrinology.

[ref-58] Xiang ZF, Sayers K (2009). Reports on seasonality of mating and birth events in wild black-and-white snub-nosed monkeys (*Rhinopithecus bieti*) at Xiaochangdu, Tibet. Primates.

[ref-59] Xiang ZF, Yang BH, Yu Y, Yao H, Grueter CC, Garber PA, Li M (2014). Males collectively defend one-male units against bachelor males in a multi-level primate society. American Journal of Primatology.

[ref-60] Yan CE, Jiang ZG, Li CW, Zen Y, Tan NN, Xia SZ (2003). Monitoring the menstrual cycle and pregnancy in the Sichuan golden monkey (*Rhinopithecus roxellana*) by measuring urinary estradiol and progesterone. Acta Zoologica Sinica.

[ref-61] Zhang SY, Liang B, Wang LX (2000). Seasonality of mating and birth in captive Sichuan Golden Monkey (*Rhinopithecus roxellana*). American Journal of Primatology.

[ref-62] Zhang YZ, Quan GQ, Zhao TG, Southwick CH (1992). Distribution of primates (except *Macaca*) in China. Acta Theriologica Sinica.

[ref-63] Zhao Qk, Deng ZY (1988). *Macaca thibetana* at Mt. Emei, China: II. Birth seasonality. American Journal of Primatology.

[ref-64] Ziegler T, Hodges K, Winkler P, Heistermann M (2000). Hormonal correlates of reproductive seasonality in wild female hanuman langurs (*Presbytis entellus*). American Journal of Primatology.

